# Krukenberg tumor in a pregnant patient with severe preeclampsia

**DOI:** 10.3892/etm.2014.1616

**Published:** 2014-03-12

**Authors:** JIE ZHANG, XINYU CHENG, CHA HAN, ZENGYAN LI, MIN WANG, YUE ZHU

**Affiliations:** 1 Department of General Surgery, Tianjin Medical University General Hospital, Tianjin 300052, P.R. China; 2 Tianjin Medical University, Tianjin Medical University General Hospital, Tianjin 300052, P.R. China; 3 Department of Obstetrics and Gynecology, Tianjin Medical University General Hospital, Tianjin 300052, P.R. China

**Keywords:** Krukenberg tumors, severe preeclampsia, color Doppler ultrasonography

## Abstract

Krukenberg tumors accompanied by gestational hypertension are rare and have a poor patient prognosis. A gravida 1, para 0 patient was submitted to Tianjin Medical University General Hospital (Tianjin, China) at 32 weeks gestation with symptoms of nausea, vomiting and hypertension. Diagnosis from the gastroscopic biopsy was of a gastric ulcer. A unilateral ovarian mass was identified with B-scan ultrasonography and magnetic resonance imaging, but was confirmed pathologically as a bilateral Krukenberg tumor. Positron emission tomography-computed tomography revealed a high radioactive uptake in the lesser curvature wall of the stomach, and postoperative pathology revealed poorly differentiated adenocarcinoma of the stomach. As Krukenberg tumors are difficult to diagnose, exhibit fast progression and have a poor clinical outcome, developing a greater understanding of Krukenberg tumors is crucial. Imaging manifestations combined with serological examination may aid in early detection, which may lead to improved patient management.

## Introduction

A Krukenberg tumor is metastatic ovarian mucin-filled signet-ring cell carcinoma (1), that exhibits fast progression and has a poor outcome. Krukenberg tumors account for 1–2% of all ovarian tumors (2) and the 5-year survival rate for a patient with a Krukenberg tumor ranges between 12 and 23.4% (3). The morbidity of pregnant patients with a Krukenberg tumor is even rarer and the survival rate is usually poor (2–5). The present study reported on a patient with gestational hypertension in addition to a progressive Krukenberg tumor. A unilateral encapsulated pedunculated solid and cystic mass with multiple nodules in the solid portion was identified by ultrasound and magnetic resonance imaging (MRI). However, pathological examination confirmed that bilateral adnexa were also involved. The tumor had three features in ultrasonography: increased quickly, had multiple nodular components in the solid portion and had a main vessel with small branches penetrating from the tumor pedicle into the solid portion.

## Case report

### Patient history

A 31-year-old female, gravida 1, para 0, was referred to Tianjin Medical University General Hospital (Tianjin, China) at 32 weeks gestation due to nausea and vomiting, with a blood pressure of 155/101 mmHg. The patient had experienced epigastric discomfort for two weeks prior to admission, however, did not see a doctor until the vomiting had persisted for three days. The patient had gained 7 kg in the last month and had previously had regular menses (4–5 days each time with a menstrual cycle of 37 days, dysmenorrhea was negative). The last menstrual period had been July 2, 2012, and the estimated date of confinement was April 9, 2013. A uric human chorionic gonadotropin (HCG) test was positive following 40 days of amenorrhea. Fetal movements were felt at four months gestation and a four dimensional ultrasound examination was normal at 28 weeks gestation. The patient had previously experienced two episodes of stomach bleeding (one year and two years ago) due to unknown reasons, but the gastroscopy examinations had appeared to be normal. The patient’s mother had a history of hypertension. Serological tests revealed an increased number of white blood cells and mildly abnormal hepatic and renal function, additionally the D-dimer levels were abnormally high (Table I). A urine test revealed that protein quantitation was 241.6 mg/dl, and positive for urobilirubin (++) and ketone (+). Written informed patient consent was obtained from the patient.

### Diagnosis

**Figure 1:**
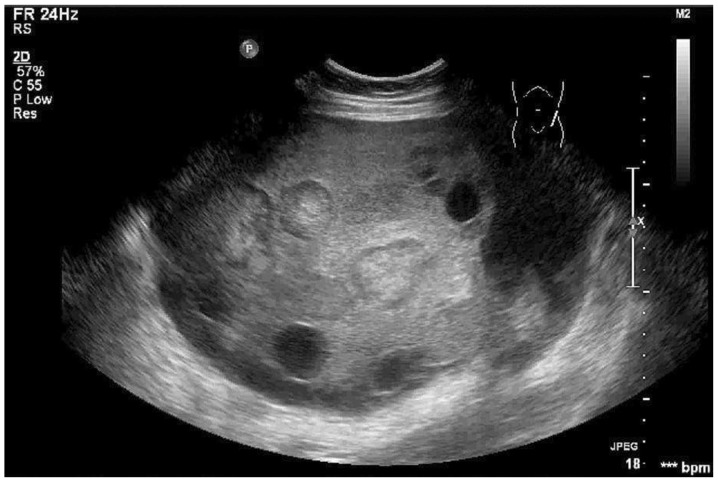
Multiple hyperechoic nodules are visible in the solid area. Multiple cysts of uneven size form the cystic portion, some of which exhibit dense punctate echo inside.

**Figure 2:**
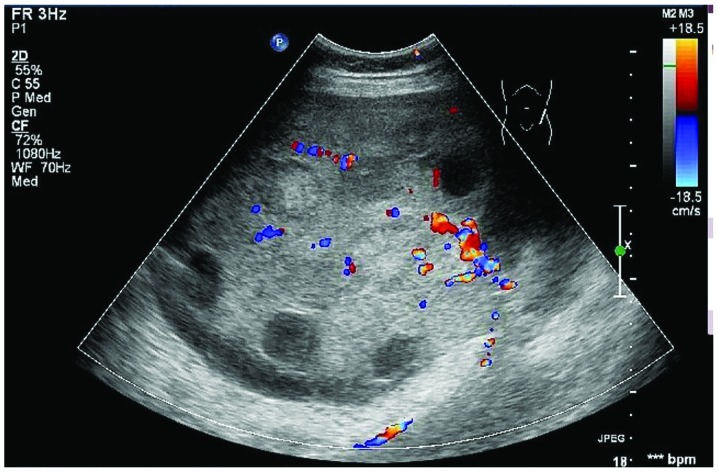
Main vessel with small branches is shown to penetrate from the tumor pedicle into the solid portion. Pulsatility index, 0.87; resistive index, 0.52; time-averaged maximum velocity, 27.63.

**Figure 3:**
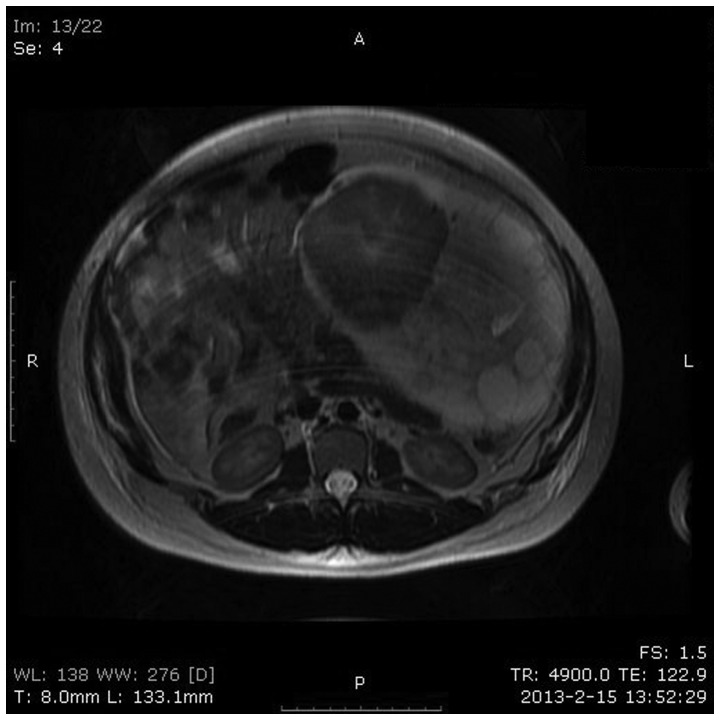
Multiple solid nodules and scattered cystic components may be observed in the mass, and a necrotic center may be demonstrated in the largest solid component.

Transabdominal color Doppler examination revealed an encapsulated pedunculated solid and cystic mass (20×18×13 cm) with irregular but clear margins on the front left of the uterus (Fig. 1). A main vessel was observed, with a pulsatility index of 0.76, a resistive index of 0.52 and a time-averaged maximum velocity of 27.63 (Fig. 2). There was a large quantity of fluid surrounding the mass. From MRI, the patient was diagnosed epithelial cancer and the imaging features were consistent with the ultrasound in terms of location, size, external configuration and internal structure (Fig. 3). Tumor markers revealed α-fetoprotein, carcinoembryonic antigen (CEA), cancer antigen 19-9, cancer antigen 125 and HCG levels to be abnormally high (Table II). Gastroscopy revealed a long ulcer lesion, 2.4 cm in length, in the posterior wall of the middle of the gastric body. The diagnosis of the biopsy was of a gastric ulcer.

### Treatment

**Figure 4:**
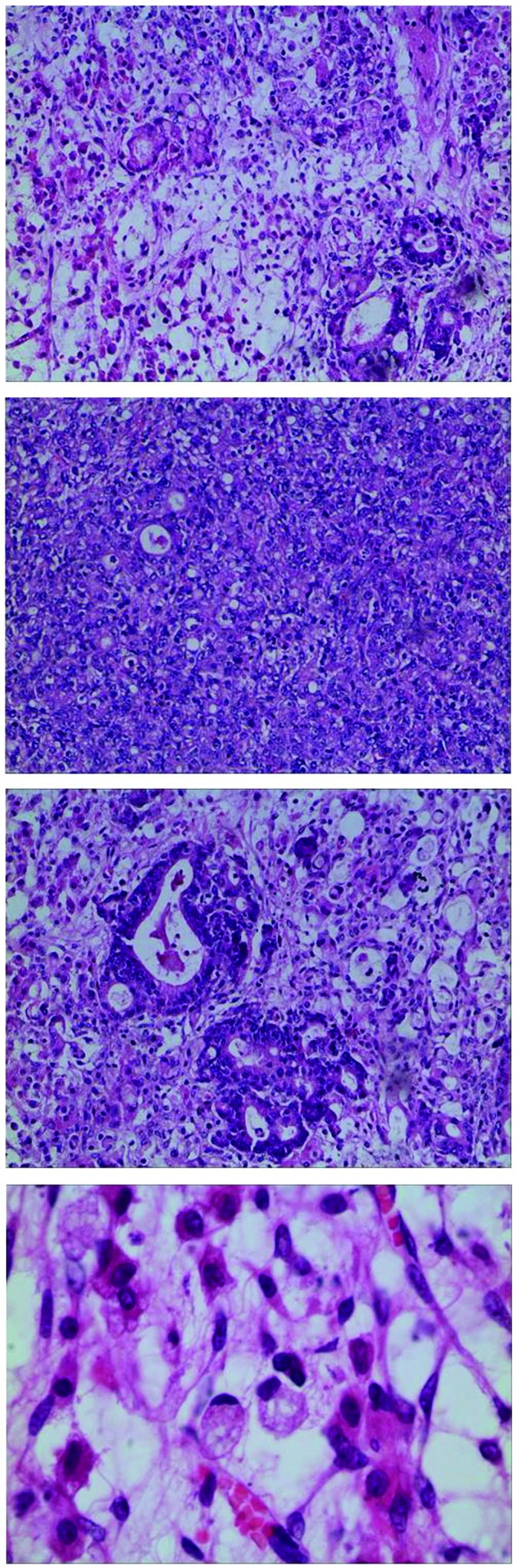
Low magnification images revealed poorly-differentiated adenocarcinoma. The gland and acinar structures have almost disappeared. Signet-ring cells may be observed at a higher magnification. Hematoxylin and eosin staining; magnification, ×100, and ×400 for the last image

**Figure 5:**
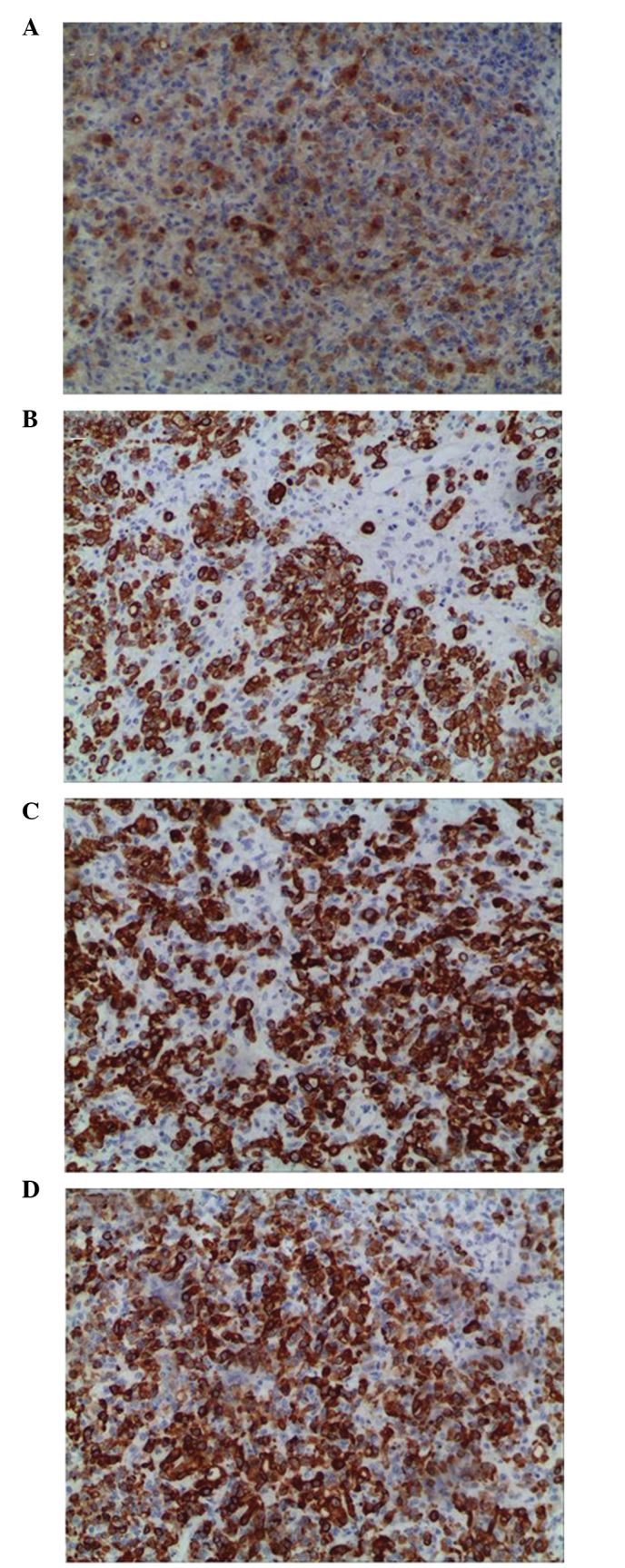
Immumohistochemistry images showing positive results for (A) CEA, (B) CK, (C) CK7 and (D) CK20. CEA, carcinoembryonic antigen; CK, cytokeratin. Immunohistochemical staining by streptavidin-perosidase; magnification, ×100.

At week 38, the patient underwent an abdominal exploration and a healthy male infant weighing 1,300 g was delivered by cesarean section, with Apgar scores of 6 and 8 at 1 and 5 min, respectively. The left uterine appendages were removed, and subsequent pathological examination of the frozen section of the left ovary revealed metastatic poorly-differentiated adenocarcinoma (Fig. 4). Subsequently, a right adnexectomy was performed. Pathological examination revealed minimal invasion of the carcinoma tissue in the right ovary, although the tissue appeared normal from the abdominal exploration and imaging observations. Macropathological analysis of the left ovary revealed multiple nodules (diameters, 0.2–5 cm) in the mass. Immunohistochemistry results indicated that CEA, cytokeratin (CK), CK7 and CK20 were positive (Fig. 5), while ascites were negative.

### Follow-up

Five days after surgery, the patient underwent a positron emission tomography-computed tomography scan. High levels of radioactive uptake were detected in the lesser curvature wall of the stomach, which indicated gastric carcinoma. The patient underwent a total gastrectomy and regional lymph node dissection combined with intraperitoneal chemotherapy. Postoperative pathology revealed poorly-differentiated adenocarcinoma with signet-ring cell carcinoma of the stomach, which infiltrated the serous membrane. In addition, a cancerous embolus was observed in the blood vessels.

## Discussion

Krukenberg tumors are a type of metastatic ovarian tumor. The primary tumor usually originates from the gastrointestinal tract, primarily from the stomach or the colon and rectum, although occasionally the tumors originate from the breast, uterus, biliary tract, pancreas and kidney (1). The bloodstream, lymphatic system and local implantation are common methods of Krukenberg tumor metastasis (6). The tumor is predominantly solid and often inflicts bilateral ovaries, with a clear border and irregular shape, which occasionally exhibits a single or multiple cystic structure. According to MRI (7) and pathology (1,6) examinations, more than half of solid tumors exhibit a random distribution of multiple nodular components. This is uncommon in primary ovarian tumors and is useful in discriminating Krukenberg tumors from primary ovarian tumors. However, sonographic observations from existing case reports of Krukenberg tumors commonly describe the tumors as roughly uniform solid masses with a strong echo, but lacking internal nodular structure (3,8). Sonographic observations in the present study revealed an encapsulated pedunculated solid and cystic mass, with multiple hyperechoic nodules in the solid portion, which was in accordance with the MRI and pathological results. Furthermore, a main vessel with small branches was observed to penetrate from the tumor pedicle into the solid portion, with high speed and low resistance. Compared with other imaging examinations, color Doppler sonography is safer, inexpensive and more convenient for diagnosing these diseases, particularly in pregnant patients (9). Thus, the sonographic observations of Krukenberg tumors are important.

The level of ovarian hormones during pregnancy and a rich blood flow contribute to tumor metastases. The most common clinical manifestations of Krukenberg tumors during pregnancy are nausea and vomiting, which are similar to symptoms often experienced during pregnancy, thus, may be neglected. As the gestational period increases and the circumference of the waist increases, the pelvic neoplasm and ascites are difficult to locate (4). Therefore diagnosis is often delayed, as demonstrated by the present case. Disease progression of Krukenberg tumors is often fast, thus, numerous patients present with metastatic carcinoma earlier than the primary tumors. If a patient has a history of primary gastrointestinal tumors or gastrointestinal symptoms, including stomach bleeding, and suffer from nausea and vomiting in the middle or later stages of pregnancy, a Krukenberg tumor may be considered as a differential diagnosis. With regard to the patient in the present case, the four dimensional ultrasound examination was normal one month previously, but a 20×18×13 cm mass was located 4 weeks later. The quick progression may correlate with severe preeclampsia, which is predominantly caused by placental hypoxia ischemia (10). Placental hypoxia may release a variety of soluble factors, leading to a high dynamic blood condition and accelerated renal blood flow, potentially promoting tumor progression.

Examining the images in the current case only located the ovarian neoplasm on the left side, but pathological diagnosis indicated that the carcinoma had also invaded the right ovary. This is due to tumor metastasis, particularly from the stomach, which is usually via a blood or lymphatic channel and leads to bilateral adnexa involvement. However, the degree of involvement varies. Certain tumors may be located by image examinations, while others cannot. Therefore, the diagnosis of a Krukenberg tumor should depend on pathological confirmation. Once unilateral lesions are located by image examination, it is important to be aware that contralateral metastasis may also exist. Thus, more care should be taken during abdominal exploration, as resecting all metastases is important to improve the patient prognosis.

In conclusion, Krukenberg tumors are difficult to diagnose, progress fast and have a poor outcome, thus, improving the understanding of Krukenberg tumors is of particular importance. When a solid and cystic mass is identified in a pregnant woman by ultrasound, with multiple hyperechoic nodules and a main vessel with small branches in the solid portion, that may be a Krukenberg tumor instead of a primary tumor, further examinations should be performed to substantiate the diagnosis and identify the source, this may help doctors to chose the best remedy and gain more survival time for the patient.

## Figures and Tables

**Table I:** Positive serological test.

Inspection item	Test result	Normal range
White blood cell, ×10^9^	19.36	4–10
Neutrophils, %	87.7	50–70
Hemoglobin, g/l	103	110–150
Serum total protein, g/l	54	63–82
Serum albumin, g/l	29	35–50
Globulin, g/l	25	26–37
Lactate dehydrogenase, U/l	456	94–250
Serum urea, mmol/l	8.0	2.5–7.1
Uric acid, mmol/l	60	140–414
Calcium, mmol/l	1.8	2.1–2.55
D-Dimer, ng/l	2107	<500
Fibrinogen, g/l	1.39	1.8–4

**Table II:** Tumor marker.

Inspection item	Test result	Normal range
α-fetoprotein, ng/ml	542.2	<8.1
CEA, ng/ml	20.64	<5
Cancer antigen 19-9, U/ml	204.01	<37
Cancer antigen 125, U/ml	>600	<30.2
HCG, mIU/ml	>10,000	<10

HCG, human chorionic gonadotropin; CEA, carcinoembryonic antigen.
